# E-cigarette school policy and staff training: Knowledge and school policy experiences with e-cigarette products among a national sample of US middle and high school staff

**DOI:** 10.1371/journal.pone.0264378

**Published:** 2022-03-16

**Authors:** Minal Patel, Emily M. Donovan, Bethany J. Simard, Barbara A. Schillo

**Affiliations:** Schroeder Institute at Truth Initiative, Washington, DC, United States of America; PLOS, UNITED KINGDOM

## Abstract

**Background:**

As youth e-cigarette use has surged in the last several years, teachers and school administrators have reported challenges addressing student use of emerging e-cigarette products on school property. While federal policy prohibits smoking in U.S. schools that receive federal funding, school e-cigarette bans only exist where states or localities have acted. Little is known about school staff experiences with implementing these relatively new policies; this study examines associations between school e-cigarette policies and trainings on school staff awareness and intervention on student e-cigarette use.

**Methods:**

A national convenience sample of 1,526 U.S. middle- and high-school teachers and administrators was surveyed in November-December 2018. Among respondents who provided their job title and indicated that they worked in a school rather than a district (n = 1,480, response rate = 97.0%), separate logistic regressions examine associations of school policies and policy training with e-cigarette awareness and intervention on student e-cigarette use.

**Results:**

Despite being the most popular e-cigarette at the time, fewer than half (47.5%) of respondents identified an image of a JUUL device as an e-cigarette. However, respondents reporting the presence of e-cigarette policies in their schools had higher odds of recognizing e-cigarettes (OR = 3.85, p<0.01), including photo recognition of JUUL (OR = 1.90, p<0.001). Respondents reporting e-cigarette policies also had higher odds of reporting intervention on student e-cigarette use (communicating with students about e-cigarette avoidance: OR = 2.32, p<0.001; reporting students had been caught using e-cigarettes at school: OR = 1.54, p<0.05). Among respondents reporting a school e-cigarette policy, those trained on the policy had higher odds of JUUL photo recognition (OR = 1.54, p<0.01). Respondents trained on e-cigarette policies also had higher odds of reporting intervention (communicating: OR = 3.89, p<0.001; students caught using e-cigarettes: OR = 2.71, p<0.001).

**Conclusions:**

As new tobacco products enter the market, school policies may be important tools to raise school personnel awareness of and intervention on emerging e-cigarette product use. However, policy adoption alone is not sufficient; policy training may further aid in recognition and intervention upon student use of e-cigarettes at school.

## Introduction

E-cigarette use among adolescents in the United States (US) has surged in recent years, with the Surgeon General declaring it an epidemic in 2018 [1]. From 2017 to 2019, past 30-day e-cigarette use among U.S. high school students increased from 11.7% to 27.5% [2]. While e-cigarette use among high school students decreased in 2020, it remains at epidemic levels with more than one in five young people currently using e-cigarettes [3]. Much of the recent increase in e-cigarette use has been attributed to the use of JUUL, a pod-based e-cigarette introduced in 2015 that gained popularity among youth through its marketing tactics that appealed to young people [4, 5]. By the end of 2018, JUUL was the most popular e-cigarette device, with two-thirds of the market share [6]. E-cigarettes like JUUL are appealing to youth for several reasons, including peer use and the ability to hide e-cigarettes from adults, which has been reported as a reason for youth initiating and experimenting with e-cigarettes [7, 8]. The popularity of JUUL among young people has been of particular concern due to its high level of nicotine, especially given that JUUL delivers nicotine at a rate similar to combustible cigarettes [9]. Although the long-term impacts of adolescent e-cigarette use are yet to be seen, addressing youth use of e-cigarettes like JUUL is critically important, as research demonstrates that nicotine is harmful to the developing adolescent brain [10, 11].

Over the last few years, there have been several accounts of teachers and school administrators across the country challenged with addressing student e-cigarette use at school [12–14]. Research has documented student use of e-cigarettes, including JUUL, on school property, including school bathrooms, staircases, and other places where students intend to hide their use [15–19]. Data from the 2019 National Youth Tobacco Survey indicate that over 60 percent of US adolescents have witnessed someone using an e-cigarette on school grounds, with the most common location being the bathroom [18, 19]. Further, although many young people indicate that e-cigarettes like JUUL appeal to them in part due to their concealability and report ease of hiding these products from adults, school personnel also report seeing students use e-cigarettes at school [12–14, 20]. Several public health and substance use experts have recognized the significance of the school environment for youth vaping interventions: the Surgeon General’s 2016 report on youth e-cigarette use identified teachers as important stakeholders in addressing youth e-cigarette use, and many school-based interventions have been developed or adapted to address youth e-cigarette use around the US, including the FDA’s Real Cost campaign, CATCH My Breath, and smokescreen [21]. Research has demonstrated that the school environment and school norms have a unique influence on students’ e-cigarette and cigarette use behaviors [22–24], and several studies indicate that both school vaping prevalence and witnessing someone using e-cigarettes on school property are associated with individual e-cigarette use [18, 19, 24, 25]. Additionally, adolescents most commonly cite “friends” as their main source of e-cigarettes [26], and report being offered or pressured into using e-cigarettes in school environments [21]. Given that U.S. adolescents spend a significant amount of time in schools, interpersonal relationships with school personnel and peers can impact student health behaviors including tobacco use [27].

In addition to the impact of peer influences and interpersonal relationships on adolescent e-cigarette use, policies play an important role in preventing and restricting youth tobacco use. In 1994, the Pro-Children Act prohibited smoking in U.S. schools that receive federal funding. School policies prohibiting tobacco, including e-cigarettes, are associated with reduced tobacco use among students; however research suggests school policies targeted at combustible cigarettes have varied greatly in terms of their comprehensiveness, clarity of rules, policy enforcement, availability of education, and prevention efforts–all of which are associated with policy impacts on tobacco use prevalence [28, 29]. Additionally, research indicates that training school staff on e-cigarette prevention and other school health interventions may improve staff knowledge and implementation of such interventions [30–33].

While federal policies to address youth e-cigarette use have been enacted in recent years, no federal policy exists to restrict e-cigarette use at schools [34, 35]. In the absence of a federal school tobacco policy that includes e-cigarettes, states, municipalities, and individual schools have become responsible for prohibiting e-cigarette use on school property [20]. Given the epidemic levels of adolescent e-cigarette use, concerns about adolescent nicotine dependence, and the influence of the school environment on tobacco use, it is important to understand how school e-cigarette policies are associated with school personnel’s knowledge, preparedness, and efforts to address student e-cigarette use. However, few studies have described school personnel experiences with policies. This study examines the potential impacts of school e-cigarette policies, policy training, and e-cigarette perceptions on school personnel e-cigarette knowledge, perceived implementation of the policy (i.e. whether they perceive students are caught using e-cigarettes), and prevention-based communication with students about e-cigarette avoidance.

## Methods

### Participants

Data were collected from a national non-probability convenience sample using an online Qualtrics panel from November-December 2018 (S1 Text). This study was approved by Advarra Institutional Review Board (Pro00030275) and all participants provided written informed consent. Participants self-identified as US teachers in middle (5th-8th grade) or high (9th-12th grade) school, or as administrators (i.e. principals, vice principals, superintendents, and other administrators) at the middle, high, or district levels (n = 1,526). Quotas were set for both middle and high school teachers for sufficient distribution to match census population estimates of the four US census regions (Northeast, Midwest, South, West). Respondents who reported working at a school district site or superintendent’s office (n = 43), rather than a school, were excluded from the analysis as it is likely they would have lower exposure to students and therefore less exposure to student vaping and related interventions to address vaping concerns. Participants missing valid job title entries (n = 3) were also excluded from the analysis, resulting in an analytic sample of n = 1,480 and a response rate of 97.0%.

### Instrumentation

#### Job title

Respondents identified as teachers, principals, vice principals, other administrators, or superintendents and indicated whether they worked within a school or a school district/superintendent’s office. Respondents were dichotomized into 1) school teachers, and 2) school administrators, or administrators who indicated that they worked within a school.

#### Current tobacco use

Respondents were categorized as current cigarette smokers if they had 1) smoked at least 100 cigarettes in their lifetime and 2) smoked at least 1 cigarette in the past 30 days. Respondents were categorized as current e-cigarette users if they had used e-cigarettes other than JUUL in the past 30 days (yes/no). Individuals were considered current JUUL users if they had used JUUL in the past 30 days (yes/no); they may have also used other e-cigarettes as well.

#### School characteristics

Enrollment was measured as a five-level ordered categorical variable indicating the total number of students enrolled in the respondents’ school (1–499, 500–999, 1000–1499, 1500–1999, 2000+). School level was measured as a three-level categorical variable indicating whether the respondent worked in a 1) middle school (or junior high school), 2) high school, or 3) combined middle (junior high)/high school. Those working in combined elementary/middle schools, combined elementary/middle/high schools, or in home schools were removed from the sample.

#### Census region

Respondents indicated which census region they resided in with the following categories: Midwest, Northeast, South, and West.

#### E-cigarette and JUUL recognition

E-cigarette text recognition was ascertained by asking participants if they had ever heard of or seen e-cigarettes. Respondents were provided with the following description: “E-cigarettes, also known as e-cigs, vapes, vape pens, modes, and tanks are devices that operate by heating a liquid solution to a high enough temperature so that it produces an aerosol that is inhaled” (yes/no). JUUL recognition was measured using two items: 1) participants were shown a JUUL device photo (S1 Fig) and asked to identify the product from a list of items (JUUL photo recognition), and 2) participants were asked if they had seen or heard of a product called JUUL (JUUL text recognition) (yes/no).

#### E-cigarette policies and training

Participants were categorized as working at schools with an e-cigarette policy if their school or district (1) had a general e-cigarette policy or (2) if they had a policy specifically about JUUL (yes, no, don’t know). Those who responded “yes” to the first question, indicating that their school had an e-cigarette policy, were asked whether they received in-service training on the policy during the past 12 months.

#### Perceived frequency students caught using e-cigarettes

Participants indicated their opinions on how often students are caught using e-cigarettes, including JUUL, on a 7-point scale. Responses were dichotomized into: ever caught (5+ times per day, 2–4 times per day, daily, at least once a week, at least once a month, less than once a month) and never caught.

#### Perceived e-cigarette problem

Participants indicated the degree to which student use of e-cigarettes (including JUUL) on school property had been a problem in the past 12 months (very serious, moderately serious, minor, and not a problem).

#### E-cigarette avoidance conversations

Respondents were asked whether they had talked to students in their school about how to avoid e-cigarette use (including JUUL use) in the past 12 months (yes/no).

### Data analysis

Univariate analyses of school and respondent characteristics, census region, e-cigarette recognition, and school e-cigarette prevention and intervention were conducted for the overall sample and by the participant’s school level (i.e., middle school, high school, and combined middle/high school). The unadjusted associations between these measures and school level were evaluated using Pearson’s chi-squared tests.

Separate logistic regression models examined the association of the presence of an e-cigarette policy with 1) e-cigarette text recognition (among non-e-cigarette users), 2) JUUL text recognition, 3) JUUL photo recognition, 4) speaking with students about e-cigarette avoidance, and 5) perceiving that students had been caught using e-cigarettes on school property. Models were adjusted for whether respondents perceived e-cigarette a problem, school enrollment, school level, job title, and respondent smoking status. All models were also adjusted for a current e-cigarette or JUUL use composite variable (no current use of JUUL or e-cigarettes; current use of e-cigarettes other than JUUL and no current JUUL use; and current use of JUUL regardless of use of other e-cigarettes) which were constructed to reduce the number of variables omitted due to collinearity. The associations of respondent e-cigarette use and JUUL use with e-cigarette and JUUL recognition were omitted due to collinearity.

Among school personnel with a school e-cigarette policy, separate logistic regression models examined the association of policy training with 1) e-cigarette text recognition (among non-e-cigarette users), 2) JUUL text recognition, 3) JUUL photo recognition, 4) speaking with students about e-cigarette avoidance, and 5) perceiving that students had been caught using e-cigarettes on school property. All models were adjusted for whether respondents perceived e-cigarette problem, school enrollment, respondent smoking status, and the current e-cigarette or JUUL use composite variable. The associations of respondent e-cigarette use and JUUL use with e-cigarette and JUUL recognition were omitted due to collinearity. In each model, observations were excluded from the analysis when missing the dependent variable or at least one covariate. Analyses were conducted in Stata 15.1. (StataCorp).

## Results

The study sample included 1,480 middle school and high school teachers and administrators; 50.7% were high school personnel, 40.7% were middle school personnel, 9.6% worked at a combined middle/high school, and most (84.4%) respondents were teachers (Table 1). Of all respondents, 11.8% were current smokers and 11.0% were current e-cigarette users (4.9% reported non-JUUL e-cigarette use, 6.1% reported JUUL use); there were no significant differences in tobacco use status by school level. Almost all (97.8%) school personnel had heard of e-cigarettes, while 71.8% recognized JUUL by name or photo. Across school levels, over 80% of school personnel reported that their school had an e-cigarette policy, but only 30.2% of those reporting a policy indicated that they had received training on the policy. Most respondents (78.7%) perceived that student e-cigarette use on school property was a problem (minor problem: 34.5%, moderate problem: 29.1%, or very serious problem: 14.2%). However, across all school levels, fewer respondents perceived that students had been caught using e-cigarettes on school property (65.0%) and even fewer (40.0%) reported speaking with students about e-cigarette prevention in the past 12 months.

**Table 1:** Characteristics of schools and school personnel by school level.

	All School Personnel	Middle School Personnel	High School Personnel	Combined Middle/ High School Personnel	Chi-square test p-values
	N = 1,480	N = 603	N = 750	N = 127	
	n (%)	n (%)	n (%)	n (%)	
School characteristics					
**Enrollment**					**<0.001**
1–499	388 (27.0)	160 (27.3)	164 (22.5)	64 (51.6)	
500–999	480 (33.4)	277 (47.3)	177 (24.3)	26 (21.0)	
1,000–1,499	227	89	119	19	
1,500–1,999	122 (8.5)	27 (4.7)	93 (12.8)	3 (2.4)	
2,000+	221 (15.4)	33 (5.6)	176 (24.1)	12 (9.7)	
**School type**					**<0.001**
Public non-charter	1,248 (84.4)	550 (91.4)	607 (80.9)	91 (71.7)	
Public charter	73 (4.9)	23 (3.8)	41 (5.5)	9 (7.1)	
Private	98 (6.6)	16 (2.7)	68 (9.1)	14 (11.0)	
Other	60 (4.1)	13 (2.2)	34 (4.5)	13 (10.2)	
**Census region**					
Midwest	359 (24.3)	136 (22.6)	186 (24.8)	37 (29.1)	0.431
Northeast	355 (24.0)	140 (23.2)	180 (24.0)	35 (27.6)	
South	429 (29.0)	185 (30.7)	216 (28.8)	28 (22.0)	
West	337 (22.8)	142 (23.5)	168 (22.4)	27 (22.3)	
Respondent characteristics					
**Job title**					**0.002**
School Teacher	1,249 (84.4)	532 (88.2)	618 (82.4)	99 (78.0)	
School Administrator	231 (15.6)	71 (11.8)	132 (17.6)	28 (22.0)	
**Current cigarette smoker**					0.717
No	1,298 (88.2)	532 (89.0)	655 (87.6)	111 (88.8)	
Yes	173 (11.8)	66 (11.0)	93 (12.4)	14 (11.2)	
**Current e-cigarette user**					
No current use of JUUL or other e-cigarettes	1308 (89.0)	530 (88.9)	666 (89.2)	112 (88.9)	0.705
Current e-cigarette use, excluding JUUL	72 (4.9)	34 (5.7)	32 (4.3)	6 (4.8)	
Current JUUL use	89 (6.1)	32 (5.4)	49 (6.6)	8 (6.3)	
**Current JUUL user**					
No	1,391 (94.0)	571 (94.7)	701 (93.5)	119 (93.7)	0.635
Yes	89 (6.0)	32 (5.3)	49 (6.5)	8 (6.3)	
E-cigarette recognition					
**Recognized e-cigarettes**					0.547
No	32 (2.2)	14 (2.3)	17 (2.3)	1 (0.8)	
Yes	1,446 (97.8)	589 (97.7)	733 (97.7)	124 (99.2)	
**Recognized JUUL by name**					**0.004**
No	474 (32.1)	222 (36.9)	213 (28.4)	39 (30.7)	
Yes	1,004 (67.9)	380 (63.1)	536 (71.6)	88 (69.3)	
**Recognized JUUL by photo**					0.743
No	775 (52.4)	318 (52.7)	387 (51.6)	70 (55.1)	
Yes	705 (47.6)	285 (47.3)	363 (48.4)	57 (44.9)	
E-cigarette prevention and intervention					
**Any e-cigarette policy**					0.370
No	129 (8.7)	63 (10.4)	57 (7.6)	9 (7.1)	
Yes	1,244 (84.1)	499 (82.8)	638 (85.1)	107 (84.3)	
Don’t know	107 (7.2)	41 (6.8)	55 (7.3)	11 (8.7)	
**Received training on e-cigarette policy**					**0.002**
No	983 (69.8)	421 (74.0)	469 (65.5)	93 (75.0)	
Yes	426 (30.2)	148 (26.0)	247 (34.5)	31 (25.0)	
**Perceived e-cigarette problem at school**					**<0.001**
Not a problem	314 (21.3)	172 (28.7)	121 (16.2)	21 (16.5)	
Minor problem	522 (35.4)	238 (39.7)	234 (31.3)	50 (39.4)	
Moderately serious	429 (29.1)	127 (21.2)	267 (25.7)	35 (27.6)	
Very serious problem	209 (14.2)	62 (10.4)	126 (16.8)	21 (16.5)	
**Frequency caught student using e-cigarettes**					**<0.001**
Never	506 (35.0)	262 (44.4)	198 (27.2)	46 (36.8)	
Ever	938 (65.0)	328 (55.6)	531 (72.8)	79 (63.2)	
**Spoke with students in past 12 months about e-cigarette avoidance**					0.669
No	876 (60.0)	362 (61.4)	441 (59.4)	73 (57.9)	
Yes	583 (40.0)	228 (38.6)	302 (40.6)	53 (42.1)	

Table 2 reports model predictors of e-cigarette recognition among all school personnel. Among all respondents, school personnel reporting an e-cigarette policy had higher odds of e-cigarette text recognition (OR = 3.73, CI = 1.65, 8.42), JUUL text recognition (OR = 2.14, CI = 1.57, 2.92), and JUUL photo recognition (OR = 1.83, CI = 1.33, 2.53) compared to respondents who reported that their school did not have an e-cigarette policy, controlling for other factors including school personnel tobacco use and school enrollment. Odds of JUUL text recognition and JUUL photo recognition generally increased as the perceived severity of student e-cigarette use increased, with those who perceived it as a very serious problem having 2.51 (CI = 1.61, 3.91) the odds of JUUL text recognition and 2.66 (1.78,3.99) the odds of JUUL photo recognition compared to those not perceiving it as a problem. Respondents who reported current JUUL use, regardless of use of other e-cigarettes, had higher odds of JUUL text recognition (OR = 4.60, CI = 2.02, 10.47) and JUUL photo recognition (OR = 1.71, CI = 1.03, 2.85) compared to non-e-cigarette users.

**Table 2:** Predictors of e-cigarette/JUUL recognition among all school personnel.

	Model 1	Model 2	Model 3
	E-cigarette recognition n = 1,257	JUUL device name recognition n = 1,406	JUUL device photo recognition n = 1,408
School has any type of e-cigarette policy			
No	REF	REF	REF
Yes	3.73** [1.65,8.42]	2.14*** [1.57,2.92]	1.83*** [1.33,2.53]
Perception of e-cigarette problem			
Not a problem	REF	REF	REF
Minor problem	1.07 [0.37,3.09]	1.57** [1.15,2.14]	1.99*** [1.45,2.73]
Moderately serious problem	0.68 [0.24,1.96]	2.05*** [1.45,2.88]	3.03*** [2.17,4.24]
Very serious problem	0.87 [0.23,3.29]	2.51*** [1.61,3.91]	2.66*** [1.78,3.99]
Enrollment			
1–499	REF	REF	REF
500–999	2.35 [0.92,6.03]	1.21 [0.89,1.65]	1.39 [0.98,1.97]
1,000–1,499	3.87 [0.85,17.58]	1.33 [0.91,1.94]	1.39 [0.98,1.97]
1,500–1,999	4.44 [0.56,34.95]	1.34 [0.83,2.17]	1.77* [1.14,2.75]
2,000+	1.69 [0.58,4.95]	1.17 [0.79,1.72]	1.24 [0.86,1.78]
School level			
High school	REF	REF	REF
Middle school	-^†^	0.77 [0.60,1.01]	1.15 [0.90,1.47]
Combined middle/high school	-^†^	1.09 [0.70,1.72]	1.15 [0.90,1.47]
Job title			
School teacher	REF	REF	REF
School administrator	0.38* [0.16,0.87]	0.75 [0.54,1.04]	0.74 [0.55,1.01]
Current smoker			
No	REF	REF	REF
Yes	1.05 [0.24,4.65]	1.07 [0.70,1.64]	0.58** [0.40,0.85]
Respondent e-cigarette use			
No current use of JUUL or other e-cigarettes	REF	REF	REF
Current e-cigarette use, excluding JUUL	-^†^	1.21 [0.68,2.18]	1.46 [0.85,2.51]
Current JUUL use	-^†^	4.60*** [2.02,10.47]	1.71* [1.03,2.85]

* p < 0.05,

** p < 0.01,

*** p < 0.001.

^†^Omitted due to collinearity.

Exponentiated coefficients; 95% confidence intervals in brackets.

Each column represents a separate logistic regression model where the predictors are the same in each model and the outcome is the variable listed at the top of the column. Model 1 examines the association between the predictors and whether respondents had heard of e-cigarettes. Model 2 examines the association between the predictors and whether respondents recognized the name “JUUL” or an image. Model 3 examine the association between the predictors and whether respondents recognized an image of the JUUL device.

Model results for school personnel intervening on student e-cigarette use are presented in Table 3. These results include odds of: 1) communicating with students about e-cigarette avoidance and 2) perceiving that students had been caught using e-cigarettes on school property. Odds of reporting communication with students were higher if respondents reported being a current JUUL user, regardless of current use of other e-cigarettes (OR = 3.13, CI = 1.72, 5.72), compared to non-e-cigarette users. Odds of reporting communication with students about e-cigarette avoidance also increased if respondents reported that their school had an e-cigarette policy (OR = 2.39, CI = 1.59, 3.60), and as the perceived severity of student e-cigarette use increased (Very serious problem: OR = 21.40, CI = 12.62, 36.27). Respondents had higher odds of reporting communication with students about e-cigarette avoidance if they reported working in a middle school versus a high school (OR = 1.44, CI = 1.09, 1.91) or if they indicated they were a school administrator versus a teacher (OR = 2.02, CI = 1.44, 2.83). Contrastingly, the perception that students were caught using e-cigarettes was lower among respondents who worked in middle schools compared with high schools (OR = 0.66, CI = 0.49, 0.89), and was not associated with respondents’ job type. School personnel had increased odds of perceiving that students had been caught using e-cigarettes on school property if they indicated that the school had an e-cigarette policy (OR = 1.40, CI = 0.97, 2.02) versus not, as perceived severity of student e-cigarette use increased (Very serious problem: OR = 27.31, CI = 15.39, 48.46), and if they reported current JUUL use (OR = 5.41, CI = 2.15, 13.59) versus non-e-cigarette users.

**Table 3:** Predictors of school personnel communication with students about e-cigarette avoidance.

	Communication with students about e-cigarette avoidance	Perception that students are caught using e-cigarettes
	n = 1,388	n = 1,376
School has any type of e-cigarette policy		
No	REF	REF
Yes	2.39*** [1.59,3.60]	1.40* [0.97,2.02]
Perception of e-cigarette problem		
Not a problem	REF	REF
Minor problem	3.67*** [2.37,5.69]	6.97*** [4.89,9.92]
Somewhat serious problem	9.98*** [6.37,15.64]	18.65*** [12.34,28.18]
Very serious problem	21.40*** [12.62,36.27]	27.31*** [15.39,48.46]
Enrollment		
1–499	**REF**	**REF**
500–999	0.73 [0.53,1.02]	1.34 [0.95,1.90]
1,000–1,499z	0.62* [0.42,0.93]	1.09 [0.72,1.65]
1,500–1,999	0.57* [0.35,0.94]	2.85*** [1.56,5.23]
2,000+	0.95 [0.63,1.43]	1.46 [0.93,2.28]
School level		
High school	REF	REF
Middle school	1.44* [1.09,1.91]	0.66** [0.49,0.89]
Combined middle/high school	1.06 [0.66,1.69]	0.78 [0.48,1.28]
Job title		
School teacher	REF	REF
School administrator	2.02*** [1.44,2.83]	0.97 [0.66,1.43]
Current smoker		
No	REF	REF
Yes	1.24 [0.82,1.88]	0.73 [0.45,1.18]
Respondent e-cigarette use		
No current use of JUUL or other e-cigarettes	REF	REF
Current e-cigarette use, excluding JUUL	1.68 [0.92,3.05]	1.64 [0.84, 3.23]
Current JUUL use	3.13*** [1.72,5.72]	5.41*** [2.15, 13.59]

Exponentiated coefficients; 95% confidence intervals in brackets.

* p < 0.05,

** p < 0.01,

*** p < 0.001.

Each column represents a separate logistic regression model where the predictors are the same in each model and the outcome is the variable listed at the top of the column. Model 1 examines the association between the predictors and whether the respondent reported ever having communicated with students about e-cigarette avoidance. Model 2 examines the association between the predictors and the perception that students are caught using e-cigarettes/JUUL, which was measured by asking school personnel how often they perceived students were caught using e-cigarettes/JUUL on school property (5+ times per day, 2–4 times per day, daily, at least once a week, at least once a month, less than once a month, and never caught). The responses were dichotomized into no (perceived that students are never caught) and yes (perceived that students are caught 5+ times per day, 2–4 times per day, once per day, at least once a week, at least once a month, and less than one month).

Model results for e-cigarette and JUUL recognition among school personnel who reported that their school had an e-cigarette policy are presented in Table 4. These respondents had increased odds of JUUL text and JUUL photo recognition if they had been trained on the school’s policy (OR = 1.68, CI = 1.24, 2.28; OR = 1.54, CI = 1.18, 1.99) compared to those not trained, and had generally increased odds as perceived severity of student e-cigarette use increased (Very serious problem: OR = 2.19, CI = 1.31, 3.66; OR = 2.22, CI = 1.40, 3.52) compared to those who did not see use as a problem. Respondents who used JUUL also had higher odds of JUUL name recognition (OR = 5.02, CI = 1.92, 13.11), but not JUUL photo recognition, compared to non-e-cigarette users. Respondents who indicated they were school administrators had lower odds than school teachers of JUUL device (OR = 0.69, CI = 0.48, 1.00) or JUUL photo recognition (OR = 0.64, CI = 0.46, 0.89). There were no associations between e-cigarette text recognition and policy training or perceptions of student e-cigarette use as a problem.

**Table 4:** Predictors of e-cigarette/JUUL recognition among school personnel working in a school with an e-cigarette/JUUL policy.

	Model 1	Model 2	Model 3
	E-cigarette recognition	JUUL device name recognition	JUUL device photo recognition
	n = 1,031	n = 1,158	n = 1,160
Had training on policy			
No training	REF	REF	REF
Training	2.92 [0.77,11.12]	1.66** [1.21,2.28]	1.55** [1.18,2.04]
Perception of e-cig problem			
Not a problem	REF	REF	REF
Minor problem	2.13 [0.51,8.86]	1.62** [1.36,3.02]	2.95*** [2.01,4.35]
Somewhat serious problem	0.95 [0.25,3.65]	2.03*** [1.35,3.02]	2.95*** [2.01,4.35]
Very serious problem	0.79 [0.15,4.09]	2.19** [1.31,3.66]	2.22*** [1.40,3.52]
Enrollment			
1–499	REF	REF	REF
500–999	1.32 [0.38,4.81]	0.90 [0.67,1.23]	0.95 [0.72,1.24]
1,000–1,499	REF	REF	REF
1,500–1,999	3.23 [0.38,27.24]	1.38 [0.81,2.36]	1.80* [1.11,2.91]
2,000+	0.95 [0.26,3.44]	0.96 [0.61,1.50]	1.29 [0.86,1.94]
School level			
High school	REF	REF	REF
Middle school	-^†^	0.75 [0.55,1.00]	1.07 [0.81,1.40]
Combined middle/high school	-^†^	1.18 [0.70,1.97]	0.94 [0.60,1.48]
Job title			
School teacher	REF	REF	REF
School administrator	0.40 [0.13,1.20]	0.69* [0.48,1.00]	0.64** [0.46,0.89]
Current smoker			
No	REF	REF	REF
Yes	1.48 [0.19,11.86]	0.99 [0.62,1.59]	0.46*** [0.30,0.70]
Respondent e-cigarette use			
No current use of JUUL or other e-cigarettes	REF	REF	REF
Current e-cigarette use, excluding JUUL	-^†^	1.28 [0.65,2.53]	1.71 [0.93,3.15]
Current JUUL use	-^†^	5.02*** [1.92,13.11]	1.63 [0.94,2.84]

* p < 0.05,

** p < 0.01,

*** p < 0.001.

^†^Omitted due to collinearity.

Exponentiated coefficients; 95% confidence intervals in brackets.

Each column represents a different logistic regression model where the predictors are the same and the outcome is the variable listed at the top of the column. All models examine respondents who indicated that their school has an e-cigarette policy. Each column represents a separate logistic regression model where the predictors are the same in each model and the outcome is the variable listed at the top of the column. Model 1 examines the association between the predictors and whether respondents had heard of e-cigarettes. Model 2 examines the association between the predictors and whether respondents recognized the name “JUUL” or an image. Model 3 examine the association between the predictors and whether respondents recognized an image of the JUUL device.

Table 5 explores predictors of intervention on student e-cigarette use among school personnel who reported that their school had an e-cigarette policy. Among these respondents, odds of communicating with students about e-cigarette avoidance generally increased if they had training on their school’s policy (OR = 3.22, CI = 2.41, 4.31), higher perceived severity of student e-cigarette use (Very serious problem: OR = 15.00, CI = 8.31,27.08), or currently used JUUL (OR = 2.51, CI = 1.30, 4.85). Respondents who reported working in a middle school and who indicated they were a school administrator had higher odds of communicating with students about e-cigarette avoidance than those working in high schools or as school teachers, respectively (OR = 1.61, CI = 1.18, 2.19; OR = 1.98, CI = 1.36, 2.88). Similar to the full sample, the perception that students were caught using e-cigarettes was lower among respondents who worked in middle schools compared with high schools (OR = 0.65, CI = 0.47, 0.91), and was not associated with respondents’ job type. Respondents with a school e-cigarette policy had higher odds of perceiving that students had been caught using e-cigarettes on school property if they had been trained on the policy (OR = 2.71, CI = 1.91, 3.84) or perceived student e-cigarette use as a problem (10.57, CI = 7.20, 15.52). Among respondent tobacco behaviors, only current JUUL use was associated with perceiving that students had been caught using e-cigarettes on school property (OR = 5.01, CI = 1.52, 13.09).

**Table 5:** Predictors of communicating with students about e-cigarette avoidance among school personnel working in a school with an e-cigarette/JUUL policy.

	Communicated with students about e-cigarette avoidance	Perception that students are caught using e-cigarettes
	n = 1,150	n = 1,136
Had training on policy		
Training	REF	REF
No training	3.22*** [2.41,4.31]	2.28*** [1.58,3.29]
Perception of e-cigarette problem		
Not a problem	REF	REF
Problem	3.08*** [1.89,5.00]	6.44*** [4.27,9.70]
Somewhat serious problem	7.39*** [4.48,12.20]	18.35*** [11.37,29.63]
Very serious problem	15.00*** [8.31,27.08]	20.07*** [10.66,37.78]
Enrollment		
1–499	REF	REF
500–999	0.74 [0.51,1.06]	1.63* [1.10,2.41]
1,000–1,499	0.60* [0.39,0.94]	1.31 [0.81,2.10]
1,500–1,999	0.63 [0.36,1.09]	2.92** [1.51,5.65]
2,000+	0.91 [0.57,1.45]	1.40 [0.84,2.33]
School level		
High school	REF	REF
Middle school	1.61** [1.18,2.19]	0.65* [0.47,0.91]
Combined middle/high school	1.32 [0.79,2.21]	0.86 [0.50,1.48]
Job title		
School teacher	REF	REF
School administrator	1.98*** [1.36,2.88]	0.98 [0.64,1.51]
Current smoker		
Yes	REF	REF
No	1.24 [0.79,1.95]	0.98 [0.64,1.51]
Respondent e-cigarette use		
No current use of JUUL or other e-cigarettes	REF	REF
Current e-cigarette use, excluding JUUL	1.25 [0.65,2.39]	1.39 [0.64,3.03]
Current JUUL use	2.51** [1.30,4.85]	4.81** [1.79,12.89]

* p < 0.05,

** p < 0.01,

*** p < 0.001.

Exponentiated coefficients; 95% confidence intervals in brackets.

Each column represents a different logistic regression model where the predictors are the same and the outcome is the variable listed at the top of the column. All models examine respondents who indicated that their school has an e-cigarette policy. Model 1 examines the association between the predictors and whether the respondent reported ever having communicated with students about e-cigarette avoidance. Model 2 examines the association between the predictors and the perception that students are caught using e-cigarettes/JUUL, which was measured by asking school personnel how often they perceived students were caught using e-cigarettes/JUUL on school property (5+ times per day, 2–4 times per day, at least once a week, at least once a month, less than once a month, and never caught). The responses were dichotomized into no (perceived that students are never caught) and yes (perceived that students are caught 5+ times per day, 2–4 times per day, once per day, at least once a week, at least once a month, and less than one month).

## Discussion

Findings from this study underscore the importance of implementing school-based e-cigarette policies to facilitate teacher and administrator recognition of e-cigarette products and subsequent intervention on student e-cigarette use. However, the existence of school e-cigarette policies alone may not be sufficient; results from this study suggest that training school personnel on such policies may further increase school personnel’s awareness of and subsequent intervention on student e-cigarette use. These findings are consistent with previous literature demonstrating the importance of training teachers on new school policies and programs [30, 31]. While it is encouraging that the vast majority of school personnel in this study reported that their school had an e-cigarette policy, fewer than half indicated that they had received training on the policy. Additionally, despite most teachers reporting that student e-cigarette use was a problem, fewer school personnel reported that students had been caught using e-cigarettes on school property and even fewer school personnel reported communicating with students about e-cigarette avoidance. This may be in part explained by the finding that fewer than half of respondents were able to identify a photo of a JUUL as an e-cigarette at a time when youth use was at epidemic levels. Further, school administrators had lower odds of JUUL device or photo recognition than teachers, indicating a gap in training. Together, these findings highlight the importance of closing the gap between existence of school-based e-cigarette policies and school personnel training, which may increase awareness about what the most popular e-cigarettes products look like and on how to appropriately intervene with students. This is especially important as the proliferation of emerging tobacco products and youth use continues to evolve in response to policy loopholes. For example, following FDA guidance that prohibits the sale of flavored cartridge-based e-cigarettes but permits flavored disposable e-cigarettes, JUUL sales have decreased, but youth use of the disposable PuffBar device has surged [36–38]. Our findings suggest that there may be a lag in school personnel recognizing new tobacco products–even those that are the most popular product among youth–highlighting the need for policies and trainings to continually assess student tobacco product use in schools. Indeed, recent reports in the popular press indicate that teachers and school administrators remain concerned and challenged by student use of e-cigarettes at school [12, 13]. Given these concerns, ensuring that school personnel have the knowledge and skills to intervene on student e-cigarette use remains important.

To date, there is limited research on the effectiveness of school e-cigarette policies and associated trainings. One longitudinal study in Ontario, Canada found that students had lower odds of using e-cigarettes if they attended a secondary school that implemented a ban on e-cigarette use on school property; however, this study did not examine the role of training in e-cigarette policy implementation [29]. Other research on school-based e-cigarette interventions has identified teacher training as an important tool to increase teacher knowledge of e-cigarettes and facilitate program implementation. Additionally, several studies highlight teacher training as an essential, yet often neglected, factor in implementing school health initiatives with fidelity [30, 39–42]. Across these studies on teacher training, researchers have identified several important training objectives, including: motivating teachers by giving background on the severity issue, the importance of their role, and incorporating teacher input; providing rationale, theories, or models that guide the intervention; and clearly communicating the responsibilities and responsible parties involved in the intervention. School administrators in our study were more likely to communicate with students on e-cigarette avoidance than school teachers–regardless of whether the school had an e-cigarette policy–likely due to procedures in policy implementation; however, it is important for teachers to also be trained on delivering e-cigarette avoidance messages. It is recommended that training takes place over time, including providing materials or ongoing technical assistance, as well as providing feedback to teachers implementing the intervention [30, 39–42].

Schools are influential environments in which youth may initiate new risk behaviors, including uptake of e-cigarettes [22, 23, 43]. Similar to the current study, previous studies have identified the school environment as one where e-cigarettes, and specifically JUUL, have been used [15, 44]. Given that youth report access to e-cigarettes from peers and that peer influences are one of the most commonly cited reasons for tobacco use, school environments need to be considered as intervention points to help youth avoid e-cigarette initiation and use [22, 23, 26]. School environments can differ by school level, as evidenced by our finding that school staff in middle schools are less likely to perceive that students are caught using e-cigarettes than high school students, likely due to the lower prevalence of use among middle school students than high school students nationally [3]. Importantly, middle school staff reported being more likely to communicate about e-cigarette avoidance than high school staff; this is promising, as tobacco prevention at an early age is associated with a lower likelihood of tobacco use [45]. However, similar communication with students on e-cigarette avoidance should exist at the high school level, particularly as emerging tobacco products continue to enter the market. Furthermore, given low levels of JUUL recognition identified in this study–especially among school administrators, results underscore the need for school personnel to be aware of youth use of emerging tobacco products and strategies to intervene on use. As the tobacco product landscape continues to evolve, it is crucial that school policies are responsive to student uptake of new products and that school personnel are equipped with the awareness and skills to help prevent and intervene on youth tobacco use.

To date, little is known about the impact of school e-cigarette policies nationally, and this study allows us to better examine the factors that are related to school personnel perceiving youth use and assisting in addressing the issue. Research indicates that broader tobacco-free school policies are most consistently associated with reduced smoking when they are comprehensive, clear, consistently enforced, and increase the availability of preventive education [28]. Additionally, research on tobacco-free school policies and similar policies, such as underage purchasing, indicate that some punitive measures such as suspension and expulsion are not effective long-term, as they may lead to student disengagement and increased tobacco use, rather than leading to changes in norms that promote abstaining from tobacco use [43, 46, 47]. School policies should therefore create an environment in which students are encouraged to abstain from e-cigarette use and school personnel are trained in identifying new devices, emerging tobacco products, school policies, and evidence-based intervention strategies. However, research is needed to determine the policy elements most effective at reducing youth use of e-cigarettes and other tobacco products.

Overall, this study found that policies were associated with higher e-cigarette awareness and intervention among school personnel and students about e-cigarettes, and that training is an important component of implementation to raise awareness of student e-cigarette use among school personnel [30, 31, 39–42]. This study highlights the need to promote a better understanding of the e-cigarette epidemic and involve all staff, including teachers and administrators, in the creation and implementation of school e-cigarette policies, including policy trainings. The combination of implementing policies and keeping teachers informed can help support school personnel in changing school environment norms. Although school personnel are likely becoming more familiar with e-cigarettes and e-cigarette policies [48], new products continue to enter the market; therefore, school personnel need ongoing support in understanding changes in youth tobacco use. Finally, the size of the school needs to be taken into consideration as individuals working in smaller schools had lower odds of perceiving student use on school grounds than larger schools, indicating that the issue may be either more prevalent at larger schools, or that teachers are more likely to perceive use in larger schools. Regardless, schools of all sizes should adopt measures to restrict e-cigarette use on school property and should be cautious and in tune with new products, as products like JUUL have become more and more discrete.

### Limitations

This research is not without limitations. First, the sample, though a national sample, is not nationally representative, as it was not possible to get a random sample of all schools in the US due to resource restrictions; hence, we did not add any weights to the sample. Additionally, this study was unable to compare perceptions of use by school personnel with actual student e-cigarette use, as these data were unable to be collected for students at the schools. Similarly, this study was unable to determine how school policy components may be related to student e-cigarette use. Future research should examine how school e-cigarette policy components, policy training components, and school personnel intervention on students’ e-cigarette use are associated with reported student e-cigarette use. Finally, data in this study were collected prior to several national events related to e-cigarettes, including the federal Tobacco T21 law, the EVALI outbreak, and national flavored e-cigarette guidance. These events may have led to greater awareness and action around youth e-cigarette use in comparison to the study period [34, 35, 49]. However, following these events, the prevalence of youth e-cigarette use was similar to the prevalence in 2018 and recent reports indicate that school personnel remain concerned about student e-cigarette use, highlighting that interventions are still needed [3, 12, 13]. Despite these limitations, this study expands an understanding of the role of school policies on intervening on student e-cigarette use.

## Conclusion

This study highlights the importance of school policies and training for school personnel in addressing the youth e-cigarette epidemic. Given the shift from physical school attendance to virtual school attendance during the COVID-19 pandemic, there is an opportunity to revamp school policies and inform teachers on how to best deal with youth e-cigarette use as in person instruction resumes. Additionally, recent shifts in the e-cigarette market away from pod-based e-cigarettes like JUUL and toward disposable and refillable cigarettes highlight the importance of training school personnel on the evolving tobacco product landscape in order to facilitate school tobacco and e-cigarette policy implementation [38]. Training staff on student e-cigarette use and policies can encourage communication with students about e-cigarette avoidance and shift norms–strategies which are encouraged and more effective long term over more punitive strategies [28, 43, 46]. Schools are encouraged to incorporate e-cigarettes into existing tobacco use policies and examine such policies to ensure that punitive actions such as suspension and expulsion are replaced with approaches such as meetings with students, cessation programs, and educational community service [43, 46].

## Supporting information

S1 Fig JUUL device photo. (DOCX)

S1 Text JUUL school survey. (DOCX)
