# Supplementary figures and images for: E-cigarette school policy and staff training: Knowledge and school policy experiences with e-cigarette products among a national sample of US middle and high school staff
Source: PLoS One. 2022 Mar 16;17(3):e0264378. doi: 10.1371/journal.pone.0264378 (PMC8926190; doi:10.1371/journal.pone.0264378)

**Supplementary Figure A**

Juul device photo


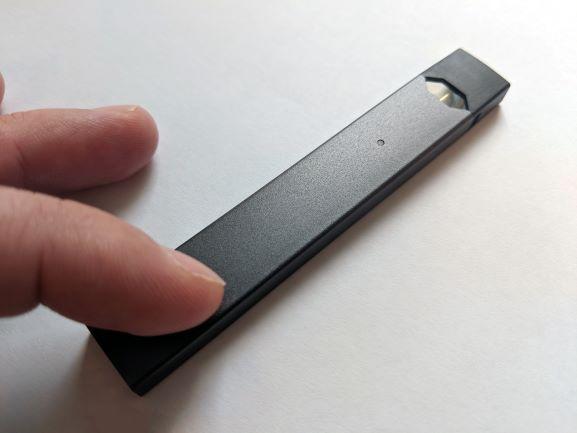

Supplement: S1 Fig — (DOCX) [file pone.0264378.s001.docx]
