# Supplementary material for: E-cigarette school policy and staff training: Knowledge and school policy experiences with e-cigarette products among a national sample of US middle and high school staff
Source: PLoS One. 2022 Mar 16;17(3):e0264378. doi: 10.1371/journal.pone.0264378 (PMC8926190; doi:10.1371/journal.pone.0264378)
Supplement: S1 Text — (DOCX) [file pone.0264378.s002.docx]

Supplemental Text A: JUUL school survey

Start of Block: Consent

ConsentText0 The purpose of this survey is to understand middle school, junior high school, and high school environments and policies. This survey is being conducted by a non-profit organization and will take no more than 10 minutes to complete.
  
 Important things to know:  There are no risks associated with taking this survey. All questions are voluntary and you may discontinue the survey at any time. We will ask information about location at which you primarily work and your professional role. However, all individual data will be anonymized, and findings will be presented in aggregate. No information will be connected to you in any way. We recommend taking the survey at a time when you can be alone if you do not want others around you to see your responses or know about your participation. There are no direct benefits to you for participation. If you have questions or concerns about your participation in this study, please contact Dr. Minal Patel at (202) 454-5906. If you have any questions about your rights as a research subject, please contact Advarra IRB at (410) 884-2900.

| 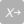 |
| --- |

Q0 Are you willing to take this survey?

- Yes (1)
- No (0)

| Page Break |  |
| --- | --- |

End of Block: Consent

Start of Block: A. Teacher/Administrator Characteristics

| 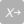 | 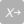 |
| --- | --- |

A1_job What is your current primary job title?

- Principal (1)
- Vice Principal (2)
- Other Administrator (3)
- Teacher (4)
- Not currently employed as a teacher/administrator (5)
- None of the above (6)

| Page Break |  |
| --- | --- |

End of Block: A. Teacher/Administrator Characteristics

Start of Block: B. Type of school

| 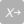 | 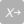 |
| --- | --- |

B2_state In which U.S. State do you work as a teacher or administrator?

▼ Alabama (1) ... Wyoming (51)

| Page Break |  |
| --- | --- |

B2_city In which city do you work as a teacher or administrator?

As a reminder, your response to this question will not be linked to your responses to the remaining survey questions.

________________________________________________________________

| Page Break |  |
| --- | --- |

| 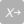 | 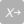 |
| --- | --- |

B3_schooltype Which of the following best describes where you primarily work?

- **Middle school** or **junior high school** (1)
- **High school** (2)
- Combined **elementary/middle/high school** or **elementary/junior high/high school** (3)
- Combined **elementary/middle school** or **elementary/junior high school** (4)
- Combined **middle/high school** or **junior high/high school** (5)
- **Home school** (6)
- **School district/superintendent's** office (7)
- I do not work at one school primarily (8)
- None of the above (9)

| Page Break |  |
| --- | --- |

End of Block: B. Type of school

Start of Block: C. Grades taught in school

Display This Question:

If B3_schooltype = Combined <b>middle/high school</b> or <b>junior high/high school</b>

| 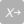 | 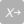 |
| --- | --- |

C4a_schoolgrades
**Please answer the subsequent questions for the school at which you primarily work.**

 What grades are taught in your school? Select all that apply.

- 4th grade (0)
- 5th grade (1)
- 6th grade (2)
- 7th grade (3)
- 8th grade (4)
- 9th grade (5)
- 10th grade (6)
- 11th grade (7)
- 12th grade (8)

| Page Break |  |
| --- | --- |

Display This Question:

If B3_schooltype = <b>Middle school</b> or <b>junior high school</b>

C4b_schoolgrades Please answer the subsequent questions for the school at which you primarily work.

What grades are taught in your school? Select all that apply.

- 4th grade (1)
- 5th grade (2)
- 6th grade (3)
- 7th grade (4)
- 8th grade (5)
- 9th grade (6)

| Page Break |  |
| --- | --- |

Display This Question:

If B3_schooltype = <b>High school</b>

C4c_schoolgrades Please answer the subsequent questions for the school at which you primarily work.

What grades are taught in your school? Select all that apply.

- 9th grade (1)
- 10th grade (2)
- 11th grade (3)
- 12th grade (4)

End of Block: C. Grades taught in school

Start of Block: D. School demographics

Display This Question:

If jobtitle = teacher

Or jobtitle = school_admin

D5a_schoolname What is the name of the school at which you primarily work?

As a reminder, your response to this question will not be linked to your responses to the remaining survey questions.

________________________________________________________________

Display This Question:

If jobtitle = district_admin

D5b_districtname What is the name of the district at which you primarily work?

As a reminder, your response to this question will not be linked to your responses to the remaining survey questions.

________________________________________________________________

|  |  |
| --- | --- |

Display This Question:

If jobtitle = teacher

Or jobtitle = school_admin

| 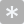 |
| --- |

D6_enrollment What is the current enrollment at your school?

________________________________________________________________

| Page Break |  |
| --- | --- |

Display This Question:

If jobtitle = teacher

Or jobtitle = school_admin

D7_schooldemographic To the best of your knowledge, what percentage of the students at your school fit the following criteria?

|  | 0 | 10 | 20 | 30 | 40 | 50 | 60 | 70 | 80 | 90 | 100 |
| --- | --- | --- | --- | --- | --- | --- | --- | --- | --- | --- | --- |

| Eligible for free or reduced-price lunch? () | 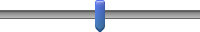 |
| --- | --- |
| Limited English Proficient (LEP)? () | 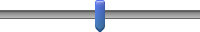 |
| Special education students? () | 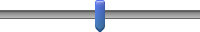 |
| Male? () | 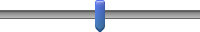 |

End of Block: D. School demographics

Start of Block: E. Teacher role

Display This Question:

If jobtitle = teacher

And B3_schooltype = Combined <b>middle/high school</b> or <b>junior high/high school</b>

| 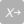 | 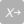 |
| --- | --- |

E8a_gradestaught What grade(s) are you currently teaching? Select all that apply.

- 5th grade (1)
- 6th grade (2)
- 7th grade (3)
- 8th grade (4)
- 9th grade (5)
- 10th grade (6)
- 11th grade (7)
- 12th grade (8)

|  |  |
| --- | --- |

Display This Question:

If jobtitle = teacher

And B3_schooltype = <b>Middle school</b> or <b>junior high school</b>

| 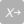 | 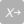 |
| --- | --- |

E8b_gradestaught What grade(s) are you currently teaching? Select all that apply.

- 5th grade (1)
- 6th grade (2)
- 7th grade (3)
- 8th grade (4)
- 9th grade (5)

Display This Question:

If jobtitle = teacher

And B3_schooltype = <b>High school</b>

| 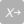 | 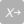 |
| --- | --- |

E8c_gradestaught What grade(s) are you currently teaching? Select all that apply.

- 9th grade (5)
- 10th grade (6)
- 11th grade (7)
- 12th grade (8)

| Page Break |  |
| --- | --- |

Display This Question:

If A1_job = Teacher

| 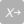 | 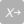 |
| --- | --- |

E9_subjects
What subject(s) are you currently teaching? Select all that apply.

- Arts and Music (1)
- English and Language Arts (2)
- English as a Second Language (3)
- Foreign Languages (4)
- Health Education/Physical Education (5)
- Mathematics/Science/Computer Science (6)
- Social Sciences (7)
- Special Education (8)
- Other (9) ________________________________________________

| Page Break |  |
| --- | --- |

End of Block: E. Teacher role


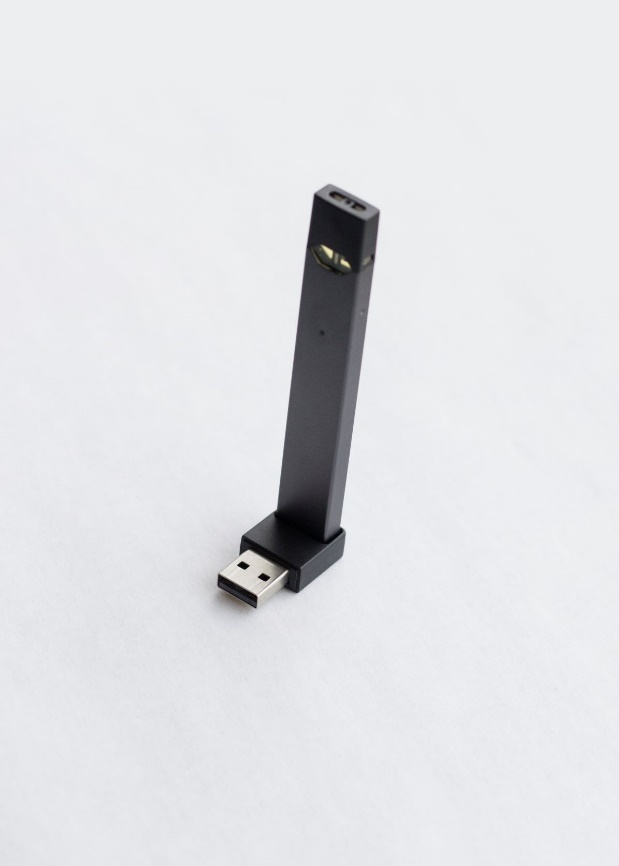
Start of Block: F. Awareness of JUUL/e-cigarettes

| 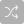 | 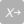 | 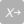 |
| --- | --- | --- |

F10_juulphotorec Which of the following best describes the device pictured above?

- Vaping device/e-cigarette (1)
- A USB storage drive (2)
- A medical device (3)
- A power bank (for charging a cell phone) (4)
- Candy (5)
- Pencil lead container (6)
- I don't know (7)


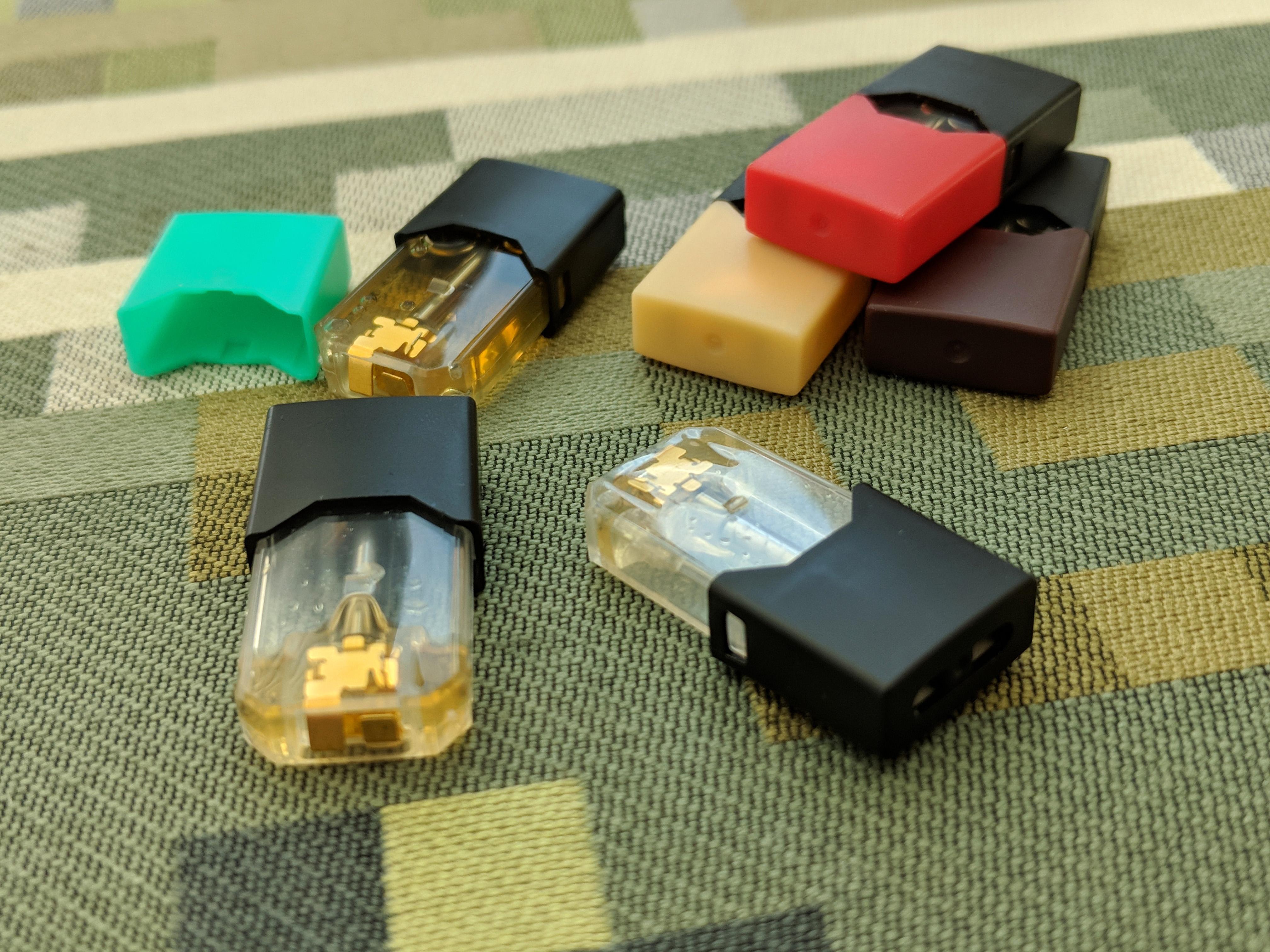


| 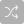 | 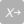 | 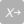 |
| --- | --- | --- |

F11_podrec Which of the following best describes the items pictured above?

- USB storage devices (1)
- Candy (2)
- E-cigarette liquid cartridges (3)
- Fuses (4)
- I don't know (5)

| Page Break |  |
| --- | --- |

| 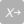 | 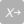 |
| --- | --- |

F12_juulnamerec Have you seen or heard of a product called JUUL (pronounced "jewel")?

- Yes (1)
- No (0)

| Page Break |  |
| --- | --- |

| 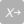 | 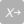 |
| --- | --- |

F13_ecigrec E-cigarettes, also known as e-cigs, vapes, vape pens, mods, and tanks, are devices that operate by heating a liquid solution to a high enough temperature so that it produces an aerosol that is inhaled. 


Have you ever seen or heard of e-cigarettes?

- Yes (1)
- No (0)

| Page Break |  |
| --- | --- |

pic3
JUUL, pictured above, is a new type of e-cigarette that is popular among young people. It heats cartridges, called pods, which contain nicotine salts and oils to create vapor.

| Page Break |  |
| --- | --- |

| 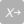 | 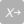 |
| --- | --- |

F14_studentschooluse In the past 12 months, where have you seen or heard of students using the following products on school property or at school-related events? Select all that apply.

|  | Cigarettes (1) | e-cigarettes (not including JUUL) (2) | JUUL (3) |
| --- | --- | --- | --- |
| Behind/beside school (beside) |  |  |  |
| In front of school (front) |  |  |  |
| Parking lot (parking) |  |  |  |
| Bathroom (bathroom) |  |  |  |
| Hallway (hallway) |  |  |  |
| Classroom (class) |  |  |  |
| Gym (gym) |  |  |  |
| Gym dressing room (locker room) (lockerrom) |  |  |  |
| Outdoor athletic facilities (field) |  |  |  |
| Other (other) |  |  |  |
| ⊗Haven't seen or heard of students using this product on school property or at school-related events (noseen) |  |  |  |

| 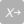 | 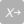 |
| --- | --- |

F15_podsatschool The is an image of JUUL pods, cartridges which the JUUL devices heat to create vapor. 


In the past 12 months, where have you seen or heard of JUUL pods on school property or at school-related events? Select all that apply.

- Behind/besides school (1)
- In front of school (2)
- Parking lot (3)
- Bathroom (4)
- Hallway (5)
- Classroom (6)
- Gym (7)
- Gym dressing room (locker room) (8)
- Outdoor athletic facilities (9)
- Other (10) ________________________________________________
- ⊗Haven't seen or heard of JUUL pods on school property or at school-related events (11)

| Page Break |  |
| --- | --- |

| 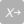 | 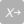 |
| --- | --- |

F16_freqstudentscaug To the best of your knowledge, how often are students caught using the following products on school property or at school-related events?

|  | 5+ times per day (7) | 2-4 times per day (6) | Daily (5) | At least once a week (4) | At least once a month (3) | On occasion (2) | Never (1) |
| --- | --- | --- | --- | --- | --- | --- | --- |
| Cigarettes (Cigarettes) |  |  |  |  |  |  |  |
| E-cigarettes (Not including JUUL) (ecigs) |  |  |  |  |  |  |  |
| JUUL (JUUL) |  |  |  |  |  |  |  |

| Page Break |  |
| --- | --- |

End of Block: F. Awareness of JUUL/e-cigarettes

Start of Block: G. School ecig/juul policies

Display This Question:

If jobtitle = teacher

Or jobtitle = school_admin

| 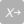 |
| --- |

G17a_ecigpolschool Has your school adopted a policy prohibiting the use of electronic vapor products, such as e-cigarettes, e-cigars, vape pipes, vaping pens, e-hookahs, and hookah pens, by students on school property or at school-related events?

- Yes (1)
- No (0)
- I don't know (99)

| Page Break |  |
| --- | --- |

Display This Question:

If jobtitle = district_admin

| 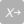 |
| --- |

G17b_ecigpoldistrict Has your district adopted a policy prohibiting the use of electronic vapor products, such as e-cigarettes, e-cigars, vape pipes, vaping pens, e-hookahs, and hookah pens, by students on school property or at school-related events?

- Yes (1)
- No (0)
- I don't know (99)

| Page Break |  |
| --- | --- |

Display This Question:

If G17a_ecigpolschool = Yes

Or G17b_ecigpoldistrict = Yes

| 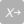 |
| --- |

G18_ecigpoljuul Does this policy specifically include JUUL?

- Yes (1)
- No (0)
- I don't know (99)

| Page Break |  |
| --- | --- |

Display This Question:

If G17a_ecigpolschool = Yes

| 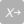 |
| --- |

G19a_ecigpolopinionS
In your opinion, is your school's e-cigarette policy...

|  | Yes (1) | No (0) |
| --- | --- | --- |
| Comprehensive? (comprehensive) |  |  |
| Clear? (clear) |  |  |
| Consistently applied? (consistent) |  |  |
| Widely publicized? (publicized) |  |  |
| Effective at preventing e-cigarette use by students on school property or at school-related events? (effective) |  |  |

| Page Break |  |
| --- | --- |

Display This Question:

If G17b_ecigpoldistrict = Yes

| 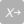 |
| --- |

G19b_ecigpolopinionD In your opinion, is your district's e-cigarette policy...

|  | Yes (1) | No (0) |
| --- | --- | --- |
| Comprehensive? (Comprehensive) |  |  |
| Clear? (Clear) |  |  |
| Consistently applied? (consistent) |  |  |
| Widely publicized? (publicized) |  |  |
| Effective at preventing e-cigarette use by students on school property or at school-related events? (effective) |  |  |

| Page Break |  |
| --- | --- |

Display This Question:

If jobtitle = teacher

Or jobtitle = school_admin

| 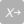 |
| --- |

G20a_schooljuulpol Has your school adopted an additional policy specifically prohibiting the use of JUUL by students on school property or at school-related events?

- Yes (1)
- No (0)
- I don't know (99)

| Page Break |  |
| --- | --- |

Display This Question:

If jobtitle = district_admin

| 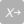 |
| --- |

G20b_districtjuulpol Has your district adopted an additional policy specifically prohibiting the use of JUUL by students on school property or at school-related events?

- Yes (1)
- No (0)
- I don't know (99)

| Page Break |  |
| --- | --- |

Display This Question:

If G20a_schooljuulpol = Yes

| 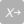 |
| --- |

G21a_Sjuulpolopinion In your opinion, is your school's JUUL policy...

|  | Yes (1) | No (0) |
| --- | --- | --- |
| Comprehensive? (comprehensive) |  |  |
| Clear? (clear) |  |  |
| Consistently applied? (consistent) |  |  |
| Widely publicized? (publicized) |  |  |
| Effective at preventing JUUL use by student's on school property or at school-related events? (effective) |  |  |

| Page Break |  |
| --- | --- |

Display This Question:

If G20b_districtjuulpol = Yes

| 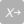 |
| --- |

G21b_Djuulpolopinion In your opinion, is your district's JUUL policy...

|  | Yes (1) | No (0) |
| --- | --- | --- |
| Comprehensive? (comprehensive) |  |  |
| Clear? (clear) |  |  |
| Consistently applied? (consistent) |  |  |
| Widely publicized? (publicized) |  |  |
| Effective at preventing JUUL use by students on school property or at school-related events? (effective) |  |  |

| Page Break |  |
| --- | --- |

Display This Question:

If jobtitle = teacher

Or jobtitle = school_admin

| 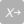 | 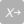 |
| --- | --- |

G22a_schoolecigpreve Has your school done any of the following as a preventive measure for e-cigarette use (including JUUL use) by students on school property or at school-related events? Select all that apply.

- Limit amount of restroom time for students (1)
- Limit number of students allowed in restroom at once (2)
- Remove restroom doors (3)
- Place devices to monitor the air in the restroom (4)
- Place devices to monitor the air elsewhere in the school (5)
- Camera surveillance near restroom (6)
- Camera surveillance elsewhere in the school (7)
- Teachers assigned to restroom duty for surveillance (8)
- Other (9) ________________________________________________
- ⊗None of the above (10)

| Page Break |  |
| --- | --- |

Display This Question:

If jobtitle = district_admin

| 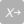 |
| --- |

G22b_Decigprevent Has your district done any of the following as a preventive measure for e-cigarette use (including JUUL use) by students on school property or at school-related events? Select all that apply.

- Limit amount of restroom time for students (1)
- Limit number of students allowed in restroom at once (2)
- Remove restroom doors (3)
- Place devices to monitor the air in the restroom (4)
- Place devices to monitor the air elsewhere in the school (5)
- Camera surveillance near restroom (6)
- Camera surveillance elsewhere in the school (7)
- Teachers assigned to restroom duty for surveillance (8)
- Other (9) ________________________________________________
- ⊗None of the above (10)

| Page Break |  |
| --- | --- |

G23_firstoffense What is the response if a student is seen with an e-cigarette (including JUUL) on school property or at school-related events for the first time? Select all that apply.

- Verbal warning (1)
- Confiscation (2)
- Contact parents (3)
- Detention (4)
- Suspension (5)
- Contact medical provider/school nurse (6)
- Provide tobacco cessation support/resources (7)
- Other (8) ________________________________________________
- ⊗There is no specific response for if a student is seen with an e-cigarette on school property or at school related events (9)

Skip To: G25_respondentviews If G23_firstoffense = There is no specific response for if a student is seen with an e-cigarette on school property or at school related events

| Page Break |  |
| --- | --- |

G24_subsequentoffens What is the response if a student is seen with an e-cigarette (including JUUL) on school property or at school-related events a subsequent time? Select all that apply.

- Verbal warning (1)
- Confiscation (2)
- Contact parents (3)
- Detention (4)
- Suspension (5)
- Contact medical provider/school nurse (6)
- Provide tobacco cessation support/resources (7)
- Other (8) ________________________________________________
- ⊗There is no specific response for if a student is seen with an e-cigarette on school property or at school-related events a subsequent time. (9)

| Page Break |  |
| --- | --- |

G25_respondentviews To what extent do you agree or disagree with the following statements?

|  | Strongly agree (1) | Somewhat agree (2) | Somewhat disagree (3) | Not agree at all (4) |
| --- | --- | --- | --- | --- |
| It is my job to intervene when I see students using e-cigarettes (including JUUL). (myjob) |  |  |  |  |
| I am willing to intervene if I see a student using an e-cigarette (including JUUL). (willingness) |  |  |  |  |
| I am confident in my ability to intervene if I see a student using an e-cigarette (including JUUL). (confident) |  |  |  |  |

| Page Break |  |
| --- | --- |

Display This Question:

If G17a_ecigpolschool = Yes

Or G17b_ecigpoldistrict = Yes

Or G20a_schooljuulpol = Yes

Or G20b_districtjuulpol = Yes

G26_enforcebarrier What do you believe are barriers to enforcing the policy on e-cigarette use/JUUL use by students on school property or at school-related events? Select all that apply.

- Lack of clarity on the policy (1)
- Lack of clarity about how the policy should be enforced (2)
- E-cigarette/JUUL products are discreet in appearance (3)
- It is difficult to pinpoint where the vapor or scent is coming from (4)
- E-cigarettes/JUUL products are addictive (5)
- Parents do not support the policy (6)
- Other (7) ________________________________________________
- ⊗There are no barriers to enforcing the policy (8)

| Page Break |  |
| --- | --- |

End of Block: G. School ecig/juul policies

Start of Block: H. School communication about juul/ecigs

Display This Question:

If jobtitle = teacher

Or jobtitle = school_admin

| 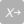 |
| --- |

H27a_schoolcom In the past 12 months, excluding any disciplinary actions, has your school done any of the following to involve or communicate with parents about e-cigarette use (including JUUL use) by students on school property or at school-related events?

- Held a Parent Teacher Association meeting or town hall meeting with parents (1)
- Sent a letter/email home to parents (2)
- Held one-on-one meetings with parents (3)
- Phone call to parents (4)
- Pre-recorded voice messages to parents (5)
- Sent messages to parents via text messages/smartphone apps (6)
- Other (7) ________________________________________________
- ⊗None of the above (8)

Display This Question:

If jobtitle = district_admin

| 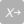 |
| --- |

H27b_districtcom In the past 12 months, excluding any disciplinary actions, has your district done any of the following to involve or communicate with parents about e-cigarette use (including JUUL use) by students on school property or at school-related events?

- Held a Parent Teacher Association meeting or town hall meeting with parents (3)
- Sent a letter/email home to parents (4)
- Held one-on-one meetings with parents (5)
- Phone call to parents (6)
- Pre-recorded voice messages to parents (7)
- Sent messages to parents via text messages/smartphone apps (8)
- Other (9) ________________________________________________
- ⊗None of the above (10)

| Page Break |  |
| --- | --- |

Display This Question:

If jobtitle = teacher

Or jobtitle = school_admin

| 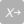 |
| --- |

H28a_schoolparents Have parents of students in your school voiced concerns to you about students' e-cigarette use (including JUUL use)?

- Yes (1)
- No (0)

Display This Question:

If jobtitle = district_admin

| 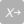 |
| --- |

H28b_districtparents Have parents of students in your district voiced concerns to you about students' e-cigarette use (including JUUL use)?

- Yes (1)
- No (0)

| Page Break |  |
| --- | --- |

Display This Question:

If jobtitle = teacher

Or jobtitle = school_admin

And G17a_ecigpolschool = Yes

Or G20a_schooljuulpol = Yes

| 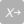 |
| --- |

H29_ecigtraining Did you receive in-service training on your school's e-cigarette use (including JUUL use) policies during the past 12 months?

- Yes (1)
- No (0)

| Page Break |  |
| --- | --- |

Display This Question:

If jobtitle = teacher

Or jobtitle = school_admin

| 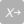 |
| --- |

H30_ecigcurriculum Is e-cigarette use prevention (including JUUL use prevention) included somewhere in your school curriculum?

- Yes (1)
- No (0)
- I don't know (99)

| Page Break |  |
| --- | --- |

Display This Question:

If jobtitle = teacher

Or jobtitle = school_admin

| 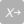 |
| --- |

H31_talkecigsstudent In the past 12 months, did you talk to students in your school about how to avoid e-cigarette use (including JUUL use)?

- Yes (1)
- No (0)

| Page Break |  |
| --- | --- |

| 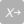 |
| --- |

H32_seenFDAad
 Have you seen these ads in school restrooms this school year?

- Yes (1)
- No (0)
- I don't know (2)

| Page Break |  |
| --- | --- |

| 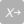 |
| --- |

H33_heardFDAad
 Have you heard students talking about these ads this school year?

- Yes (1)
- No (0)
- I don't know (99)

| Page Break |  |
| --- | --- |

End of Block: H. School communication about juul/ecigs

Start of Block: I. Ecig/JUUL priority and concern

| 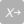 |
| --- |

I34_facconcern In your opinion, how concerned is your staff about e-cigarette use (including JUUL use) by students on school property or at school-related events?

- Very concerned (3)
- Somewhat concerned (2)
- Not at all concerned (1)

| Page Break |  |
| --- | --- |

| 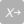 |
| --- |

I35_facpriority In your opinion, how much of a priority is it to your staff to address e-cigarette use (including JUUL use) by students on school property or at school-related events?

- Very high priority (5)
- Somewhat high priority (4)
- Medium priority (3)
- Somewhat low priority (2)
- Very low priority (1)

| Page Break |  |
| --- | --- |

| 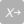 |
| --- |

I36_respconcern
How concerned are you about youth e-cigarette use (including JUUL use) by students on school property or at school-related events?

- Very concerned (3)
- Somewhat concerned (2)
- Not at all concerned (1)

| Page Break |  |
| --- | --- |

| 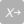 |
| --- |

I37_ecigproblem To what extent, if any, has e-cigarette use (including JUUL use) by students been a problem on school property or at school-related events during the past 12 months?

- Very serious problem (4)
- Moderately serious problem (3)
- Minor problem (2)
- Not a problem (1)

| Page Break |  |
| --- | --- |

| 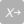 |
| --- |

I38_ecigharm Compared to cigarettes, do you think that e-cigarettes (including JUUL) are...

- Much more harmful to a person's health compared to cigarettes (5)
- Slightly more harmful to a person's health compared to cigarettes (4)
- Equally harmful to a person's health compared to cigarettes (3)
- Slightly less harmful to a person's health compared to cigarettes (2)
- Much less harmful to a person's health compared to cigarettes (1)

| Page Break |  |
| --- | --- |

| 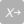 |
| --- |

I39_ecigaddiction Compared to cigarettes, do you think that e-cigarettes (including JUUL) are...

- Much more addictive compared to cigarettes (5)
- Slightly more addictive compared to cigarettes (4)
- Equally addictive compared to cigarettes (3)
- Slightly less addictive compared to cigarettes (2)
- Much less addictive compared to cigarettes (1)

| Page Break |  |
| --- | --- |

End of Block: I. Ecig/JUUL priority and concern

Start of Block: J. Participant Smoking status

| 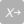 |
| --- |

J40_100cigs Have you smoked at least 100 cigarettes in your entire life?

- Yes (1)
- No (0)

| Page Break |  |
| --- | --- |

Display This Question:

If J40_100cigs = Yes

| 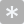 |
| --- |

J41_cigpast30 During the past 30 days, on how many days did you smoke cigarettes?

________________________________________________________________

| Page Break |  |
| --- | --- |

| 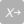 |
| --- |

J42_everJUUL Have you ever used a JUUL, even one or two times?

- Yes (1)
- No (0)

| Page Break |  |
| --- | --- |

Display This Question:

If J42_everJUUL = Yes

| 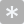 |
| --- |

J43_juulpast30 During the past 30 days, on how many days did you use JUUL?

________________________________________________________________

| Page Break |  |
| --- | --- |

| 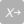 |
| --- |

J44_everecig Have you used an e-cigarette other than JUUL (refers to an e-cigarette or vaping device, such as NJOY, Blu, or tank system/box mod vaporizer) even one or two times?

- Yes (1)
- No (0)

| Page Break |  |
| --- | --- |

Display This Question:

If J44_everecig = Yes

| 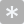 |
| --- |

J45_ecigpast30 During the past 30 days, on how many days did you use e-cigarettes other than JUUL?

________________________________________________________________

| Page Break |  |
| --- | --- |

Display This Question:

If If cig past 30 Text Response Is Greater Than 0

| 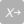 |
| --- |

J46_cigschoolprop Have you ever smoked cigarettes on school property or at school-related events?

- Yes (1)
- No (0)

| Page Break |  |
| --- | --- |

Display This Question:

If If JUUL past 30 Text Response Is Greater Than 0

Or e-cigarette past 30 Text Response Is Greater Than 0

| 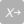 |
| --- |

J47_ecigschoolprop Have you ever used an e-cigarette (including JUUL) on school property or at school-related events?

- Yes (1)
- No (0)

| Page Break |  |
| --- | --- |

End of Block: J. Participant Smoking status

Start of Block: K. Participant Demographics

| 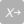 |
| --- |

K48_gender Are you...?

- Female (1)
- Male (0)
- Other (3) ________________________________________________

| Page Break |  |
| --- | --- |

| 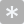 |
| --- |

K49_age What is your age?

________________________________________________________________

| Page Break |  |
| --- | --- |

K50_race Which one or more of the following categories describes your race?

- White/European American (1)
- Black/African American (2)
- American Indian/Alaskan Native/Native American (3)
- Asian/Asian American (4)
- Pacific Islander/Native Hawaiian (5)
- Other race (6) ________________________________________________

| Page Break |  |
| --- | --- |

| 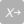 |
| --- |

K51_ethnicity Are you of Hispanic or Latino origin, such as Latin American, Mexican, Puerto Rican, or Cuban?

- Yes, of Hispanic or Latino origin (1)
- No, not of Hispanic or Latino origin (0)

| Page Break |  |
| --- | --- |

Display This Question:

If jobtitle = teacher

Or jobtitle = school_admin

| 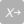 |
| --- |

K52_Stownsize
Would you say that the population of the city or town in which your school is located is...

- 50,000 or more people (3)
- At least 2,500 and less than 50,000 people (2)
- Less than 2,500 people (1)

| Page Break |  |
| --- | --- |

Display This Question:

If jobtitle = district_admin

| 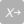 |
| --- |

K53_Dtownsize Would you say that the population of the city or town in which your district is located is...

- 50,000 or more people (3)
- At least 2,500 and less than 50,000 people (2)
- Less than 2,500 people (1)

| Page Break |  |
| --- | --- |

Display This Question:

If jobtitle = teacher

Or jobtitle = school_admin

K54_schooltype In what type of school do you currently work? Select all that apply.

- Public school (1)
- Public charter school (2)
- A magnet school or a school with a special program emphasis, e.g., science/math school, performing arts high school, talented/gifted school, foreign language immersion school (4)
- Special education: a school that primarily serves students with disabilities (5)
- Vocational/technical: a school that primarily serves students being trained for occupations (6)
- Alternative: a school that offers a curriculum designed to provide alternative or nontraditional education, not clearly categorized as regular, special education, or vocational (7)
- Private (independent) (8)
- Private (religiously affiliated) (9)
- Other (11) ________________________________________________

| Page Break |  |
| --- | --- |

Display This Question:

If jobtitle = teacher

| 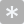 |
| --- |

K55_yrsteach By the end of this school year, how many years will you have been teaching altogether? Do not include substitute or student teaching.

________________________________________________________________

| Page Break |  |
| --- | --- |

Display This Question:

If jobtitle = teacher

| 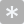 |
| --- |

K56_yrscurrentschool By the end of this school year, how many years will you have been teaching at your current school?

________________________________________________________________

| Page Break |  |
| --- | --- |

Display This Question:

If jobtitle = school_admin

Or jobtitle = district_admin

| 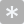 |
| --- |

K57_yrsadmin By the end of this school year, how many years will you have been a school or district administrator altogether?

________________________________________________________________

| Page Break |  |
| --- | --- |

Display This Question:

If jobtitle = school_admin

Or jobtitle = district_admin

| 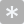 |
| --- |

K58_yrsposition By the end of this school year, how many years will you have been at your current position?

________________________________________________________________

End of Block: K. Participant Demographics
